# Study protocol for a phase I investigator-initiated clinical trial of E7820 in Japanese patients with unresectable solid tumours: CIRCUS trial (NCCH2303)

**DOI:** 10.1093/jjco/hyag031

**Published:** 2026-03-01

**Authors:** Mao Okada, Jun Sato, Chikako Funasaka, Noboru Yamamoto, Tetsuya Sasaki, Masayuki Yokoyama, Akinobu Hamada, Toshihiko Doi, Hiroyuki Mano

**Affiliations:** Department of Experimental Therapeutics, National Cancer Center Hospital, 5-1-1 Tsukiji, Chuo-ku, Tokyo 104-0045, Japan; Department of Experimental Therapeutics, National Cancer Center Hospital, 5-1-1 Tsukiji, Chuo-ku, Tokyo 104-0045, Japan; Department of Experimental Therapeutics, National Cancer Center Hospital East, 6-5-1 Kashiwanoha, Kashiwa, Chiba 277-8577, Japan; Department of Experimental Therapeutics, National Cancer Center Hospital, 5-1-1 Tsukiji, Chuo-ku, Tokyo 104-0045, Japan; Clinical Research Support Office, National Cancer Center Hospital, 5-1-1 Tsukiji, Chuo-ku, Tokyo 104-0045, Japan; Clinical Research Support Office, National Cancer Center Hospital, 5-1-1 Tsukiji, Chuo-ku, Tokyo 104-0045, Japan; Division of Molecular Pharmacology, National Cancer Center Research Institute, 5-1-1 Tsukiji, Chuo-ku, Tokyo 104-0045, Japan; Department of Experimental Therapeutics, National Cancer Center Hospital East, 6-5-1 Kashiwanoha, Kashiwa, Chiba 277-8577, Japan; Division of Cellular Signaling, National Cancer Center Research Institute, 5-1-1 Tsukiji, Chuo-ku, Tokyo 104-0045, Japan

**Keywords:** E7820, RNA splicing factors, xenograft model antitumor assays, phase I, Japan

## Abstract

E7820, a sulphonamide-type anticancer agent, was developed to inhibit angiogenesis by suppressing integrin α2 expression. Although early-phase trials outside Japan established 100 mg/day as the maximum tolerated dose in monotherapy, no objective responses were observed, and further development was not planned. Subsequent studies revealed that E7820 promotes DCAF15-mediated degradation of the splicing factor RBM39 and acts as a molecular glue, providing a novel mechanism and rationale for clinical evaluation. Preclinical screening using patient-derived xenograft models demonstrated antitumour activity in biliary tract and endometrial cancers and in tumours with homologous recombination repair gene alterations. Based on these findings, the CIRCUS trial, a multicentre investigator-initiated phase I study, was initiated to assess the safety, tolerability, and preliminary efficacy of E7820 in Japanese patients with unresectable solid tumours. If the proof of concept is demonstrated in biomarker-defined cohorts, E7820 may be repositioned for selected patients, providing insights into the development of previously intractable compounds.

## Teaser

Based on E7820’s novel mechanism and preclinical activity in specific tumour subsets, a Japanese investigator–initiated multicentre phase I trial was launched to re-evaluate its safety, tolerability, and preliminary efficacy.

## Introduction

Advanced or recurrent malignant tumours that cannot be curatively resected are associated with poor prognoses. Epidemiological surveys in Japan have reported that ~980 000 cases of cancer occur annually, of which 18.8% (~350 000 patients per year) have distant metastases [1]. The 5-year relative survival rate for malignancies with distant metastases is reported to be below 20% [1], highlighting the urgent need for further therapeutic development for patients with unresectable advanced or recurrent cancers.

### E7820

E7820 was initially developed by Eisai Co., Ltd., as a sulphonamide-type anticancer agent that inhibits tumour angiogenesis by suppressing the expression of integrin α2 [2].

A phase I trial targeting malignant tumours was conducted overseas (E7820-A001-102 trial, NCT00078637) [3]. In this trial, the starting dose regimen was defined as 10 mg of E7820 administered orally once daily (QD) for 28 days, with a 7-day treatment break included only in Course 1. The tolerability and safety of E7820 were evaluated using a 3 + 3 dose-escalation design at doses of 10, 20, 40, 70, 100, and 200 mg/day. No dose-limiting toxicities (DLTs) were observed in four patients treated with 100 mg/day E7820. However, at 200 mg/day, two of six patients experienced DLTs, including anaemia, neutropenia, thrombocytopenia, abdominal pain, and vaginal bleeding. Subsequently, an additional cohort of 13 patients was administered 100 mg/day E7820. Among the 17 patients treated with this dose, only one DLT was reported.

Based on these results, the maximum tolerated dose (MTD) and recommended phase II dose were determined to be 100 mg QD for 28 days per cycle.

The most frequently observed adverse events (AEs) included Grade 1–2 nausea, fatigue, diarrhoea, and anaemia. Grade 3–4 haematologic toxicities were also observed but were manageable with supportive care. Although 29 of 37 enrolled patients were evaluated for efficacy, no objective responses were observed in the E7820-A001-102 trial. Incidentally, patients with biliary tract or endometrial cancer were not enrolled in this trial.

E7820 is rapidly absorbed following oral administration, achieving mean maximum plasma concentrations (*C*_max_) ranging from 0.19 μg/ml at a dose of 10 mg/day to 1.86 μg/ml at 200 mg/day within 2.17–5.33 h postdose (*t*_max_). Both *C*_max_ and the area under the concentration–time curve (AUC) increased linearly up to a dose of 70 mg/day; however, a nonlinear increase was observed in the dose range of 70–200 mg/day. The mean AUC_0–24_ values ranged from 1.2 μg·h/ml (10 mg/day) to 21.6 μg·h/ml (200 mg/day), and the mean terminal half-life of E7820 was between 5.6 and 8.6 h.

A separate phase I trial (E7820-E044-110 trial [4]) was conducted in patients with unresectable solid tumours to determine the MTD of E7820 administered orally twice daily (BID). This study consisted of two parts: Part A (food-effect study) and Part B (MTD determination for BID dosing). In Part A, the primary objective was to evaluate the effect of a high-fat meal on the oral bioavailability of E7820 (50 mg). Participants were randomized to receive a single 50 mg dose under fasting conditions or immediately after a high-fat breakfast on Day 1, followed by crossover dosing after a 7-day washout period. Although E7820 showed high pharmacokinetic variability (AUC CV: 29%–137%), administration after a high-fat meal caused only a slight delay in absorption (tmax: 4 h vs. 3 h under fasting conditions). The geometric mean ratios (postprandial/fasting) of *C*_max_ and AUC were 1.12 and 1.07, respectively, with 90% confidence intervals including 1, indicating no clinically relevant food effect. Therefore, E7820 can be administered irrespective of food intake. The primary objective of Part B was to determine the MTD of E7820 for BID dosing. MTD was defined as the highest dose at which no more than one of six patients experienced DLT. Two dose levels were evaluated: at 50 mg BID, no DLTs were observed in three patients; at 60 mg BID, two of seven patients experienced DLTs (febrile neutropenia and neutropenia). Consequently, the MTD for BID dosing was determined to be 50 mg. Similar to the previous trial, the best overall response was stable disease, and no objective responses were observed.

Recently, E7820 has been shown to function as a molecular glue that facilitates the interaction between DCAF15—a substrate receptor of the CUL4 E3 ubiquitin ligase complex—and the splicing factor RBM39 (also known as CAPERα) [5–7]. This interaction promotes selective ubiquitination and proteasomal degradation of RBM39. Loss of RBM39 disrupts RNA splicing fidelity and has been implicated in the suppression of cancer cell proliferation, providing a novel rationale for the clinical investigation of E7820 [5–7].

### Background of target population selection

At the National Cancer Center (NCC) in Japan, a patient-derived xenograft (PDX) library, referred to as the J-PDX Library [8], has been established using tumour samples derived from Japanese patients with cancer. As of April 2023, this library comprised ~600 PDX models across various cancer types. Since 2021, NCC and Eisai Co., Ltd., have conducted a research project, supported by the Cyclic Innovation for Clinical Empowerment (CiCLE) programme of the Japan Agency for Medical Research and Development, to evaluate the antitumour efficacy of compounds in Eisai’s development pipeline using rare and refractory cancer models from the J-PDX Library. The CiCLE programme aims to consolidate Japan’s strengths through industry–academia–government collaboration, thereby establishing a fundamentally innovative research foundation. Its objectives include conducting research and development that precisely addresses clinical needs, accelerating the clinical application of pharmaceuticals, medical devices, regenerative medicine products, and related medical technologies and fostering an environment that strongly promotes open innovation and the growth of ventures in the field of medical research and development. As part of this initiative, it was decided to re-evaluate the efficacy of E7820, the mechanism of action of which has been elucidated in recent years.

By April 2023, drug screening for E7820 was performed on 48 PDX models, including 12 pancreatic, 12 biliary tract, 10 gastric, 10 endometrial, 2 lung, 1 ovarian, and 1 breast cancer models.

In these PDX models, efficacy screening was performed by oral administration of E7820 at doses of either 100 or 200 mg/kg for 21 consecutive days. The antitumour effect was assessed by calculating the T/C ratio using the following formula:


\begin{align*} \varDelta \mathrm{T}=\,&\left(\mathrm{Final}\kern0.17em \mathrm{tumour}\kern0.17em \mathrm{volume}\kern0.17em \mathrm{in}\kern0.17em \mathrm{the}\kern0.17em \mathrm{treatment}\kern0.17em \mathrm{group}\right)\nonumber\\&\hbox{--} \left(\mathrm{Initial}\kern0.17em \mathrm{tumour}\kern0.17em \mathrm{volume}\kern0.17em \mathrm{in}\kern0.17em \mathrm{the}\kern0.17em \mathrm{treatment}\kern0.17em \mathrm{group}\right) \end{align*}

\begin{align*} \varDelta \mathrm{C}=\,&\left(\mathrm{Final}\kern0.17em \mathrm{tumour}\kern0.17em \mathrm{volume}\kern0.17em \mathrm{in}\kern0.17em \mathrm{the}\kern0.17em \mathrm{control}\kern0.17em \mathrm{group}\right)\nonumber\\&\hbox{--} \left(\mathrm{Initial}\kern0.17em \mathrm{tumour}\kern0.17em \mathrm{volume}\kern0.17em \mathrm{in}\kern0.17em \mathrm{the}\kern0.17em \mathrm{control}\kern0.17em \mathrm{group}\right) \end{align*}

$$ \mathrm{T}/\mathrm{C}=\left(\varDelta \mathrm{T}/\varDelta \mathrm{C}\right)\times 100 $$

T/C represents the relative change in tumour volume in the treatment group compared with the control group; lower T/C values indicate greater tumour shrinkage.

A previous study [9] presented a waterfall plot of T/C values, illustrating the antitumour efficacy according to cancer type following the administration of E7820 at 100 mg/kg in each PDX model.

Among the PDX models, negative T/C values—indicating tumour shrinkage—were observed in 8 of 12 (67%) biliary tract cancer models and in 6 of 9 (67%) endometrial cancer models, suggesting a strong antitumour effect in these cancer types.

To identify biomarkers associated with E7820-induced tumour shrinkage, whole-exome sequencing was performed on PDX models. Abnormalities in homologous recombination repair (HRR) genes, which are critical for DNA repair pathways, were frequently observed in models that responded to E7820. Notably, tumour shrinkage was detected in models harbouring mutations in *BRCA1*, *BRCA2,* or *ATM*, suggesting that these mutations may serve as potential biomarkers for predicting E7820 efficacy [9].

These results provide a rationale for re-evaluating the safety and efficacy of E7820 in cancers characterized by specific histological subtypes and biomarkers that are predictive of treatment response.

Based on these findings, the CIRCUS trial, a multicentre investigator-initiated phase I study designed to evaluate the safety and tolerability of E7820 in Japan, was initiated.

In addition, this study aims to assess the efficacy of E7820 in patient populations expected to derive clinical benefit, as suggested by the results of efficacy evaluations using PDX models.

## Patients and methods

### Study design of the CIRCUS trial

We designed a multicentre phase I investigator-initiated clinical trial of E7820 in Japanese patients with unresectable solid tumours (the CIRCUS trial; NCCH2303) to evaluate the tolerability and safety of E7820 in this population. The CIRCUS trial will be conducted jointly at the NCC Hospital and NCC Hospital East. The study protocol was approved by the Institutional Review Board of NCC in June 2024 (T5246).

**Figure 1:**
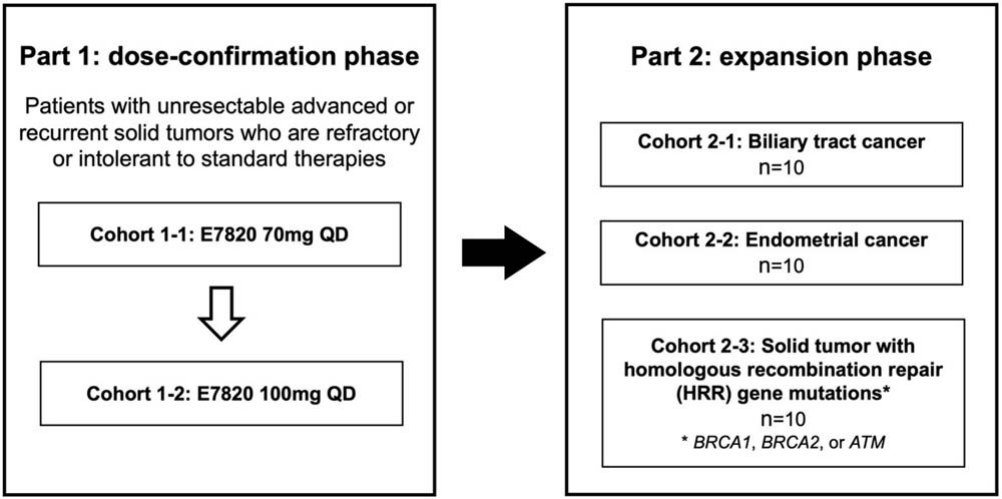
Schema of the CIRCUS trial.

The study comprises two phases: a dose-confirmation phase (Part 1) and an expansion phase (Part 2) (Fig. 1).

The dose-confirmation phase (Part 1) is designed to evaluate the tolerability and safety of E7820 in Japanese patients with advanced or recurrent solid tumours that are refractory or intolerant to standard therapeutic regimens, regardless of gene mutation status. In the dose-confirmation phase (Part 1), a 3 + 3 dose-escalation design will be used to determine the recommended dose (RD).

In the expansion phase (Part 2), efficacy and safety data will be collected at the RD determined in Part 1. Based on prior PDX preclinical studies that showed E7820-induced tumour shrinkage in biliary tract and endometrial cancers, irrespective of HRR gene status, as well as in tumours harbouring HRR mutations (*BRCA1*, *BRCA2*, and *ATM*), the following cohorts will be established: Cohort 2–1 (biliary tract cancer), Cohort 2–2 (endometrial cancer), and Cohort 2–3 (solid tumours with HRR gene mutations, such as *BRCA1*, *BRCA2*, or *ATM*).

### Eligibility criteria

#### Inclusion criteria

Table 1 provides an overview of the eligibility criteria. Eligible patients must have histologically or cytologically confirmed solid tumours that are unresectable, advanced, or recurrent. These tumours must be those for which no standard therapy is available or for which standard treatments have proven ineffective or intolerable. Patients must also have an Eastern Cooperative Oncology Group performance status of 0 or 1 and adequate organ function.

**Table 1:** Eligibility criteria.


**a) Inclusion criteria**	
Histologically or cytologically confirmed malignant solid tumour	
Unresectable, advanced, or recurrent solid tumour	
No standard treatment available or standard therapy has proven ineffective or intolerable	
Eastern Cooperative Oncology Group performance status of 0 or 1	
Expected survival of at least 3 months from the time of enrolment	
At least 18 years of age	
At least one measurable lesion identified on contrast-enhanced computed tomography or magnetic resonance imaging	
Patients must not have previously received treatment with E7820.	
Clinical laboratory tests performed within 14 days before enrolment must demonstrate adequate organ function Neutrophil count ≥1500/mm^3^ Haemoglobin ≥9.0 g/dl Platelet count ≥10 × 10^4^/mm^3^ Creatinine ≤1.5 mg/dl Total bilirubin ≤1.5 mg/dl Aspartate aminotransferase ≤75 (150) U/L (if liver metastases are present) Alanine aminotransferase ≤105 (210) U/L in males and ≤ 57.5 (115) U/L in females (if liver metastases are present)	
SpO_2_ ≥ 92% on room air	
Females of childbearing potential must agree to use effective contraception from the time of providing informed consent until 30 days after the final dose of the study drug and must also agree to refrain from breastfeeding during this period. Male patients are required to use contraception for the same duration.	
All patients must be capable of providing written informed consent and of complying with all study requirements.	
**Cohort 2–1: Biliary tract cancer** Unresectable, advanced, or recurrent biliary tract cancer, which includes intrahepatic cholangiocarcinoma, hilar cholangiocarcinoma, distal cholangiocarcinoma, ampullary carcinoma, or gallbladder cancer	
**Cohort 2–2: Endometrial cancer** Unresectable, advanced, or recurrent endometrial cancer	
**Cohort 2–3: Solid tumour with homologous recombination repair gene mutations** Solid tumour with *BRCA1*, *BRCA2*, or *ATM* gene mutation	

**b) Exclusion criteria**	
Hypersensitivity to sulphonamide derivatives	Patients with a history of unstable ischemic disease, including ischaemic heart disease, myocardial infarction, or other unstable cardiac conditions, within 3 months prior to enrollment
Anticancer treatments, including chemotherapy, molecularly targeted therapy, or immunotherapy within 4 weeks before enrolment	Require anticoagulation or antiplatelet therapy
Any other investigational or unapproved drugs within 4 weeks before enrolment	Active haemoptysis or gastrointestinal bleeding within 3 weeks before enrolment
Underwent major trauma or significant surgical procedures within 4 weeks before enrolment	Active multiple primary cancers, except for cancers that have been completely resected, gastrointestinal mucosal carcinomas that have been curatively treated, or other malignancies with no recurrence for >5 years
Patients who have not recovered to grade 1 or higher from adverse events related to prior treatments, excluding peripheral neuropathy, alopecia, and endocrine disorders controlled with hormone replacement therapy	Left ventricular ejection fraction <50% on echocardiography
Systemic steroid-based immunosuppressive therapy equivalent to ≥10 mg/day of prednisolone within 2 weeks before enrolment	Clinically significant electrocardiogram abnormalities, including QT prolongation with a QTc (Fridericia’s correction) >480 ms
Pregnant, potentially pregnant, or lactating women	Interstitial lung disease or pulmonary fibrosis
Symptomatic brain metastases, carcinomatous meningitis, or progressive central nervous system disorders	Psychiatric disorders or symptoms that interfere with daily functioning or study participation
Spinal metastases requiring radiotherapy or surgical intervention	Antipsychotic drug use or suicide attempts within the past 2 years
Clinically significant pericardial, pleural, or ascitic effusions requiring treatment	Drug or alcohol dependence or abuse within the past 2 years
Patients who had a positive test result for human immunodeficiency virus, hepatitis B virus, or hepatitis C virus	Pulmonary dysfunction requiring active treatment
Active infections requiring systemic treatment	Investigator deems medically or otherwise unfit to participate in the study

#### Additional criteria for expansion-phase cohorts

In the biliary tract cancer cohort in the expansion phase, in addition to meeting the general inclusion criteria described above, patients must have been diagnosed with unresectable, advanced, or recurrent biliary tract cancer, including intrahepatic cholangiocarcinoma, hilar cholangiocarcinoma, distal cholangiocarcinoma, ampullary carcinoma, or gallbladder cancer.

In the endometrial cancer cohort, patients must have unresectable, advanced, or recurrent endometrial cancer.

In the cohort of solid tumours with HRR gene mutations, eligible patients must have a solid tumour harbouring a *BRCA1*, *BRCA2*, or *ATM* gene mutation, as identified via either a cancer gene panel test (including liquid biopsy) such as FoundationOne® CDx or FoundationOne® Liquid CDx or a *BRCA*1/2 gene test such as BRACAnalysis®, performed as part of reimbursed or evaluated medical treatment in Japan.

#### Exclusion criteria

Table 1 summarises the exclusion criteria. Patients with a history of haemorrhagic disorders within 3 weeks before enrolment, those receiving anticoagulant therapy or immunosuppressive agents, and those with a history of interstitial lung disease will be excluded.

#### Study procedures

In the dose-confirmation phase (Part 1), E7820 will be administered at different doses, depending on the cohort. In Cohort 1–1, the participants will receive 70 mg of E7820 orally QD, whereas in Cohort 1–2, the participants will receive 100 mg of E7820 orally QD. Each treatment course is defined as 28 days.

In the E7820-A001-102 study [3], no events identified as DLTs were reported at 100 mg QD (although at the time of manuscript publication, such events had been considered DLTs, a re-evaluation performed during the preparation of the clinical study report determined, based on causality with the investigational drug and the clinical course, that they did not meet the criteria for DLTs). At 200 mg QD, DLTs were observed in two of six patients, whereas no DLTs were observed at doses of ≤70 mg QD. Furthermore, no clinically significant serious AEs or deaths were reported at doses of ≤70 mg QD. As the safety of E7820 in the Japanese population has not yet been evaluated, and based on the findings obtained from the E7820-A001-102 trial, the starting dose in the present study was set at 70 mg QD, which is one dose level lower than the MTD and RD of 100 mg QD established in the E7820-A001-102 trial. The next dose-escalation level was set at 100 mg QD, and no evaluation of tolerability at doses exceeding 100 mg QD was planned.

Initially, three participants will be enrolled in Cohort 1–1. If no DLTs are observed in the three participants, the study will proceed to Cohort 1–2. If one of the three participants experiences a DLT, an additional three participants will be enrolled in the same cohort. If one or fewer DLT is observed among the six participants after this additional enrolment, the study will proceed to Cohort 1–2. Conversely, if DLTs are observed in two or more of the first three participants or in two or more of the six participants in Cohort 1–1, the cohort will be considered to have exceeded the MTD, and the study will not proceed to Cohort 1–2.

The same evaluation process will be applied to Cohort 1–2. If no DLTs are observed in the first three participants, the dose administered in Cohort 1–2 will be determined as the RD. If one of the three participants experiences a DLT, an additional three participants will be enrolled. If one or fewer DLT is observed among the six participants after additional enrolment, the dose in Cohort 1–2 will be confirmed as the RD. However, if two or more of the initial three participants or two or more of the six participants in Cohort 1–2 experience DLTs, the cohort will be considered to have exceeded the MTD, and enrolment will be terminated. In this case, the dose administered to Cohort 1–1 will be established as the RD.

Once the RD is determined, the study will proceed to the expansion phase.

In the expansion phase (Part 2), E7820 will be orally administered QD at the RD established in the dose-confirmation phase (Part 1). In Part 2, safety will be evaluated, and exploratory assessments of efficacy—including objective response rate (ORR), progression-free survival (PFS), and overall survival (OS)—will be performed.

The expansion phase will target biliary tract cancer, endometrial cancer, and solid tumours harbouring HRR gene mutations.

The tumour types and candidate genes to be included in the expansion phase may be reviewed by the Coordinating Committee and the Trial Coordination Office based on the results of efficacy evaluations from the PDX models and may be added before the initiation of the expansion phase.

#### Definition of dose-limiting toxicity

A DLT is defined as an AE related to E7820 that occurs during Course 1 (28 days). The details of the DLT definitions are presented in Table 2. AEs will be graded based on the Common Terminology Criteria for Adverse Events (CTCAE) version 5.0 [10], Japan Clinical Oncology Group (JCOG) version [11].

**Table 2:** Definition of dose-limiting toxicity.

**Haematologic toxicity**	1. Grade 4 neutropenia for ≥8 days Grade 4 neutropenia requiring treatment with granulocyte colony-stimulating factor
	2. Febrile neutropenia
	3. Grade 3 thrombocytopenia for **≥**8 days Grade 3 thrombocytopenia accompanied by bleeding Grade 3 thrombocytopenia requiring platelet transfusion Grade 4 thrombocytopenia
	4. Grade 3 anaemia requiring red blood cell transfusion Grade 4 anaemia
	Grade 4 adverse events
**Nonhaematologic toxicity**	1. Clinically significant grade 3 adverse events (excluding diarrhoea, nausea, and vomiting that resolve to grade 1 or lower ≤7 days with appropriate treatment)
	2. Clinically significant grade 3 or higher laboratory abnormalities

In addition, if an adverse event related to E7820 results in a total administered dose during course 1 that is <75% of the planned dose, it will be considered a dose-limiting toxicity (DLT).

If treatment is discontinued owing to an adverse event not related to E7820 or if <75% of the planned dose is administered in the absence of a DLT, the case will not be subject to DLT evaluation. In such cases, the Trial Steering Committee or Trial Coordination Office will review the situation and consider enrolling a replacement participant.

#### Endpoints and outcome measures

The primary endpoint is the incidence of DLT.

The clinical hypothesis for the dose-confirmation phase of this study is that the RD of E7820 for Japanese patients is 100 mg/day, administered orally for 28 consecutive days. Because there are no existing data on the administration of E7820 in Japanese patients, tolerability will be evaluated at two dose levels: 70 mg/day (Cohort 1–1) and 100 mg/day (Cohort 1–2). A minimum of 3 and a maximum of 12 participants will be enrolled in the dose-confirmation phase.

Tolerability will be assessed based on the incidence of DLTs observed during the DLT evaluation period, which is defined as treatment course 1 (28 days of E7820 administration).

The starting dose will be 70 mg/day, and the DLT evaluation will begin with three participants.

Secondary endpoints include PK, pharmacodynamics (PD), ORR, duration of response (DoR), disease control rate (DCR), PFS, OS, and the incidence of AEs.

PK/PD analyses will be performed using the pharmacokinetic analysis set (those with appropriate PK data collection) to evaluate the plasma concentrations and pharmacokinetic parameters of E7820 as well as their correlations with efficacy and safety. PK analyses will be performed using noncompartmental analysis with the Phoenix® NLME software (Certara, St. Louis, MO, USA).

Tumour response will be assessed according to the Response Evaluation Criteria in Solid Tumors v1.1 (JCOG version) [12]. Imaging studies for efficacy assessment (computed tomography or magnetic resonance imaging) will be performed every 8 weeks until Week 24 after the initiation of the protocol treatment and every 12 weeks thereafter.

The ORR is defined as the proportion of patients whose best overall response is complete response (CR) or partial response (PR), as determined by the investigators. The DoR is defined as the period from the first confirmed CR or PR until disease progression or death and will be evaluated in the full analysis set (FAS: patients who received at least one dose, excluding those with evident protocol violations). The DCR is the proportion of patients in the FAS with the best overall response of CR, PR, or stable disease. PFS is defined as the time from enrolment to disease progression or death from any cause, whereas OS is defined as the time from enrolment to death from any cause. AE incidence is defined as the proportion of patients in the safety analysis set (SAS: those who received at least one dose) experiencing each event, with grading based on the worst severity per CTCAE v5.0-JCOG.

### Statistics

#### Analysis method

Primary analysis will be performed after completing the DLT assessment in the dose-confirmation phase, and the RD of E7820 will be determined. A standard 3 + 3 design will be used. Escalation from Cohort 1–1 to Cohort 1–2 will occur if DLTs are zero in three patients or one or fewer in six patients; otherwise, the continuation of the trial will be reviewed. In Cohort 1–2, if DLTs are one or fewer in six, 100 mg will be confirmed as the RD, and the expansion phase will proceed. The Trial Steering Committee will evaluate the validity of the DLTs at each milestone.

Safety analysis will focus on the frequency and proportion of DLTs. The 95% CI will be calculated using the Clopper–Pearson method. For efficacy, the ORR will be assessed using a binomial test (H0: ORR ≤ 5%) with one-sided 5% significance level. The CIs (90% and 95%) will be calculated for each cohort. Subgroup and supplementary analyses will be performed based on tumour type, genetic alterations, and prior treatments using FAS, SAS, and all enrolled populations.

PFS and OS will be analysed using Kaplan–Meier estimates, with Greenwood’s formula and the Brookmeyer–Crowley method applied to CIs. Additional analyses will include waterfall, spider, and swimmer plots; compliance (dose intensity) summaries; DoR; and time-to-response using both descriptive statistics and Kaplan–Meier estimates.

#### Sample size

In the dose-confirmation phase (Part 1), the tolerability of E7820 will be evaluated using a 3 + 3 design. A minimum of 3 and a maximum of 6 participants will be enrolled in each cohort, resulting in a total planned enrolment of at least 3 and up to 12 participants across the entire dose-confirmation phase.

In the expansion phase (Part 2), patients with advanced or recurrent solid tumours that are refractory or intolerant to standard therapy will be enrolled. In addition to further safety evaluations, the antitumour effect will be assessed using the ORR as a secondary endpoint. The statistical assumptions for efficacy include a threshold ORR of 5%, an expected ORR of 45%, a one-sided significance level of 5%, and 80% power. Based on these assumptions, the required number of evaluable patients per cohort was calculated to be nine using a binomial test. To account for possible ineligible cases or patients who did not receive treatment, the planned enrolment per cohort was set at 10 participants.

The threshold ORR was based on previous reports indicating that phase I trials for solid tumours after standard treatment typically yield ORRs of 4.9%–6.2% [13, 14]. A response rate exceeding this threshold would support the efficacy of E7820 in the study population. Furthermore, recently approved therapies for tumours with driver mutations have shown ORRs of ~35%–45% [15–17]; thus, an expected ORR of 45% was considered appropriate.

The planned enrolment period is 1 year for the dose-confirmation phase and 2 years for the expansion phase.

#### Monitoring

Monitoring will be performed through on-site visits involving direct inspection of source documents as well as via telephone, web-based communication, and e-mail to confirm that the trial is being conducted safely and appropriately at each participating institution and that the reliability of the data is adequately maintained. When information that may significantly affect the conduct of the trial or the reliability of the data is obtained, the monitor will prepare a monitoring report and submit it to the principal investigator and head of the institution.

#### Ethical considerations

This study will be conducted in compliance with the study protocol, the Declaration of Helsinki, Article 80-2 of the Pharmaceuticals and Medical Devices Act, the Ministerial Ordinance on Good Clinical Practice for Drugs (Ordinance of the Ministry of Health and Welfare No. 28 of 27 March 1997) and its amendments ‘GCP Ordinance’, as well as relevant notifications.

## Conclusion

In the CIRCUS trial, the tolerability and safety of E7820 are being evaluated in Japanese patients with unresectable advanced or recurrent solid tumours that are refractory or intolerant to standard therapies.

E7820 was originally developed as a sulphonamide-based anticancer agent that inhibits tumour angiogenesis by suppressing the expression of integrin α2. However, no objective responses were observed in previous clinical trials conducted outside Japan, and further drug development was considered unfeasible at that time. Recently, E7820 has been identified as a molecular glue that facilitates the interaction between DCAF15 and the splicing factor RBM39, leading to selective proteasomal degradation of RBM39. In parallel, pharmacological evaluation using PDX models has identified tumour types and genetic alterations in which E7820 may exert antitumour activity. In light of this newly elucidated mechanism of action and the results from PDX studies, we initiated this investigator-initiated clinical trial to re-evaluate the tolerability, safety, and preliminary efficacy of E7820 in Japanese patients with unresectable solid tumours.

Drug development has previously advanced through repositioning based on the identification of novel mechanisms of action or therapeutic targets [18, 19]. Given the identification of novel mechanisms of action and therapeutic targets, we aim to re-evaluate the efficacy of E7820. If a proof-of-concept can be established through biospecimen analyses, this approach may not only enable the repositioning of agents previously considered difficult to develop but also contribute to the development of future therapeutic agents. This strategy has the potential to provide valuable insights into the evolving paradigms of drug discovery and development.

Patient enrolment in the CIRCUS trial began in September 2024, and the study is currently ongoing.

## jRCT registration number

This trial was registered at the Japan Registry of Clinical Trials as jRCT2031240210.
